# 

**Published:** 1915-10

**Authors:** 


### CONTENTS.

ORIGINAL COMMUNICATIONS—

Sane Indications of the Uses of Medicinal Baths and Exercise—By Theodore Toepel, M. D., Atlanta, Ga. 313

The Post-Operative Results in Nine Cases of Gastro-Enterostomy, with one Case in Which More Than Two-Thirds of the Stomach was Resected for Cancer—By Gaston Torrance, M. D., Birmingham, Ala. 319

Scopolamine and Morphine in Labor—A Clinical Study of Twenty-One Cases—By J. L. Campbell, M. D., F. A. C. S., Atlanta, Ga. 326

Vernal Catarrh—By Dr. G. D. Ayer, M. D., Atlanta, Ga. 331

Regular Meeting of the Fulton County Medical Society, October 21, 1915—By Dr. Allen H. Bunce 332

EDITORIAL 336

SELECTIONS AND ABSTRACTS 340

BOOK REVIEWS 362

